# Improving Activity of *Lycium Barbarum.* Polysaccharide on Depressive Mice Induced by Reserpine

**DOI:** 10.22037/ijpr.2019.1100763

**Published:** 2019

**Authors:** Rui Zhao, Bing Qiu Master, Baoling Ma Master, Yaping Cai

**Affiliations:** a *Department of Pharmaceutical Engineering, College of Life Science & Biotechnology, Heilongjiang August First Land Reclamation University, Daqing High-Tech Industrial Development Zone, 163319, P. R. China.*; b *Department of Gastroenterology, Heilongjiang Province Hospital, 82 Zhongshan Road, Harbin, 150036, P. R. China.*; c *Department of Physical education, Hebei Normal University of Science and Technology, 360 Hebei Street, Qinhuangdao 066004, P. R. China.*; d *Department of Pharmaceutical Engineering, College of Life Science & Biotechnology, Heilongjiang Bayi Agricultural University, Daqing High-Tech Industrial Development Zone, 163319, P. R. China.*

**Keywords:** Lycium barbarum, Polysaccharide, Depressive disorder, Antioxidation, Apoptosis

## Abstract

Depressive disorder will be the second highest disease burden worldwide, which will impair life quality, reduce productivity, and increase disability and mortality. *Lycium barbarum.* polysaccharide (LBP) is the main active fraction purified from* Lycium barbarum.* The aim of this study was to evaluate the potential therapeutic effects of LBP on depressive mice induced by reserpine, as well as the relevant mechanisms. The antidepressant effect of LBP was investigated by open field test (OFT), forced swimming test (FST), tail suspension test (TST), and antagonism of reserpine hypothermia and ptosis in mice. In addition, we examined the oxidative status and antioxidation power of striatum in both control and depressive mice with or without LBP treatment. To explore the mechanism of LBP on regulating antioxidants in the depressive mice, we detected the expression level of Bcl-2 and poly (ADP ribose) polymerase (PARP) in striatum of mice by western blotting. The results showed that administration with LBP for 4 consecutive weeks significantly increased locomotor activity, reduced the duration of immobility, and antagonized hypothermia and ptosis in mice induced by reserpine. Also, LBP treatment was able to reduce the lipid peroxidation (LPO) production, and enhance the antioxidation effect of the striatum in depressive mice. Furthermore, LBP inhibited the decreased extent of the apoptotic suppressors, Bcl-2 and PARP, which were markedly decreased after treatment with reserpine. The above results indicated that LBP possess antidepressant activities, probably via its powerful antioxidative properties and then decreased the apoptosis of striatum neuron.

## Introduction

Depressive disorder will be the second highest disease burden worldwide, which will impair people’s quality of life, reduce productivity, and increase disability and mortality (1). Depression is associated with decreased monoaminergic neurotransmitter levels in the brain (2). Our previous experimental results have showed that the levels of dopamine, serotonin, and norepinephrine were reduced in depressive mice. Dopamine is mainly found in the striatum. The striatum serves as a central hub to regulate the motor, psychiatric, and cognitive functions of the brain. Dopamine, produced mainly from projection neurons in the substantia nigra pars reticulata (SNc), is believed to exert its primary actions in the mediumspiny neurons (MSNs) of the striatum by acting on dopamine receptors. A large number of studies have suggested that the abnormal dopamine reward circuit of midbrain-striatum in the dopamine system on the edge of the midbrain was an important depression pathologic mechanism (3-6). Various antidepressants, including tricyclic antidepressants, monoamine oxidase inhibitors, and noradrenaline (NA) reuptake inhibitors, are widely applied in clinical. Unfortunately, many of the antidepressants are non-specific and cause severe side effects (7, 8). Therefore, searching for new alternative strategies for the prevention and treatment of depressive disorder is essential. *Lycium barbarum.* polysaccharide (LBP), the active ingredient extracted from *Lycium barbarum.*, has been found bioactivities such as enhancing the body′s immune and antioxidant (9,10). We have previously shown that LBP is heteropolysaccharides and contained different carbohydrate compositions (11). In this study, we investigated the potential therapeutic effects of LBP to prevent the depression in the mice induced by reserpine and relevant mechanisms.

Oxidative stress, which is defined as a disturbance in the balance between the production of reactive oxygen species (ROS) and antioxidant defense systems, has been showed to play a role in the pathogenesis of neuropsychiatric disorders (12). A body of preclinical studies has demonstrated that the inhibition of oxidative stress may contribute to the therapeutic effects of some antidepressant drugs (13-15). However, a possible link between the antioxidant enzyme activity and antidepressant effect of LBP has not yet been investigated.

In the present study, we assessed the antidepressant effect of LBP and its mechanisms by means of behavioral and pharmacological procedures. The effect of LBP on the antioxidant in striatum has been scarcely investigated. Given this background, the primary objective of the present study was to investigate the possible mechanism of LBP against depressive disorder resulting from apoptosis of striatal neurons mediated by oxidative stress in mice. The experimental results may provide comprehensive, scientific evidence for LBP as a suitable dietary natural antidepressant agent.

## Experimental

*Drugs and Reagents*

The fruits of *Lycium barbarum.* were collected in the Ningxia Hui Autonomous Region which was the well-known production area of *Lycium barbarum* in China, and were authenticated at the Agricultural college of Northwest A&F University. A specimen (NO.20140609) was deposited in the herbarium of the Botany Department. Isolation, purification, and identification of LBP were based on our previous published work (11).

Reserpine (methyl reserpate 3, 4, 5-trimethoxybenzoic acid ester; Sigma, Mumbai) was dissolved in glacial acetic acid and then diluted to afinal concentration of 0.1% acetic acid with distilled water. Kits for the assays of superoxide dismutase (SOD), glutathione peroxidase (GSH-Px) and catalase (CAT) were purchased from the Nanjing Jiancheng Bioengineering Institute (Nanjing, China). Anti-Bcl-2 and anti-PARP antibodies were purchased from Santa Cruz Biotechnology, Inc. Anti-β-actin antibody was purchased from Sigma Chemical Co. All other reagents and chemicals were of the highest purity grade available.

*Animals and treatments*

Six-week-old male or female C57BL/6 mice (average weight, 25-30 g) were provided by the Experimental Animal Center of Hayida Medical University. The animals were treated according to the National Institute of Health Guide for the Care and Use of Laboratory Animals and further approval for their experimentation was obtained from the Animal Ethics Committee of the university. The animals were housed in an animal room at 22 ± 2 ℃ and 50 ± 10% relative humidity and had free access to laboratory chow and tap water. The mice were adapted to an inverse 12:12-h light-dark cycle. The depressive model mice were induced by administration of reserpine (2 mg/kg, s.c. daily) for 15 consecutive days followed by administration with LBP once daily for 4 weeks. In the LBP groups, the mice were treated by intragastric administration with LBP (80 mg/kg·d, the optimal dosage chosen according to our previous study) dissolved in NaCl 0.9% solution and the model group mice received NaCl 0.9% solution. In the positive control group, the mice were treated by subcutaneous injection with amitriptyline (10 mg/kg·d). In the morning of the day 28, after mice were administrated with LBP or amitriptyline for 30 min, the behavioural experiments were conducted. The brain samples were harvested immediately 1 h after the measurement of the behavior paradigms for antioxidant capacity analysis. 

*Open field test (OFT)*

The assessment of the possible effects of LBP on the locomotor activity was carried out as previously described (16) with slight modifications. Briefly, the mice were placed in a square container (100×100×30 cm) with bottom divided into 25, equal sections. Each subject was placed in the center of the open field, and the walking routes and the number of grid crossings of mice was measured for 4 min.

*Forced swimming test (FST)*

The FST was performed according to published methods with minor modification (17). Briefly, the mice were individually placed in an open cylindrical container containing 19 cm of water at 25 ± 1℃ (diameter 10 cm, height 25 cm). The mice were considered immobile when they made only the movements necessary to keep their head above water. The immobility times of the mice during the final 4 min of the 6-min test were recorded and analyzed.

*Tail suspension test (TST)*

The TST was measured according to the method described by Steru *et al. *(18) as a facile means of evaluating potential antidepressants. Briefly, the mice were individually suspended by tail with a clamp 50 cm above the floor by adhesive tape placedapproximately 1 cm from the tip of the tail. The duration of the test was 6 min, and immobility was determined for the last 4 min of the test. The mice were considered immobile only when they hung passively and completely motionless.

*Reserpine antagonism test*

The reserpine antagonism test was performed according to the method described previously (19, 20). The rectal temperature was measured with a digital thermometer. 

The degree of ptosis was rated according to the following rating scale: 0, eyes open; 1, one-quarter closed; 2, half closed; 3, three-quarters closed; 4, completely closed.

*Assessment of lipid peroxidative (LPO) indices*

The mice were sacrificed 1 h after behavioral quantification. The brain was quickly removed and the part of striatum was further dissected out. The dissected out striatum tissue was rinsed with isotonic saline and weighed, and then it was homogenized with 0.1 N HCl. A 10% (w/v) tissue homogenate was prepared in a 0.1 M phosphate buffer (pH 7.4). Lipid peroxide concentration was measured by the thiobarbituric acid reactive substance (TBARS) assay (21).

*Measurement of antioxidant enzymes*

1 g striatum tissues were homogenated. The 10% preparation of tissue homogenate was centrifuged with 3,000 rpm at 4 ℃for 15 min. The supernatant was collected for determination of SOD, GSH-Px, and CAT. The activities of SOD, GSH-Px, and CAT were measured using commercial kit. The manipulation was progressed strictly according to the kit instruction manual. 

*Western blot*

The Proteins of the striatum tissues of the mice were separated by 15% sodium dodecyl sulfate-polyacrylamide gel electrophoresis (SDS-PAGE), after which the resolved proteins were transferred to nitrocellulose membranes for 2 h. Each membrane was incubated separately with primary antibody: anti-Bcl-2, or anti-PARP antibody (Santa Cruz Biotechnology, CA, USA), overnight at 4℃. The membranes were then washed with washing buffer and incubated with horseradish peroxidase-conjugated secondary antibodies at room temperature for 1 h. After washing, the protein bands were visualized by using an enhanced chemiluminescence (ECL) system. Densitometric analysis of the western blots was performed by using a GS-670 Imaging Densitometer (BioRad) and Molecular Analyst Software (version 1.3).

*Statistical analysis*

All the results were expressed as the mean ± S.D. *P*-values of less than 0.05 were considered to be significant. Statistical analysis was performed by one-way analysis of variance (ANOVA). All the grouped data were statistically evaluated with SPSS 13.0 software. Statistical significance of differences between two groups was determined using the post hoc test.

## Results

*Characterization of LBP*

Structure analysis of LBP was based on our previous study (11). Briefly, LBP was identified to be a homogeneous polysaccharide component, which showed a single symmetrical peak following Sephadex G-100 gel chromatography. The MW of LBP was 33,867 Da and retention time was 8.257 min by HPLC. In addition, the monosaccharide composition of LBP was analyzed by paper chromatography and revealed the presence of six spots, corresponding to galactose, glucose, rhamnose, arabinose, mannose, and xylose respectively. LBP had two absorption peaks at 199 and 260 nm in the UV spectrum, indicating the presence of polysaccharide and protein. According to the IR spectrum, the purified LBP displayed a broadly stretched, intense peak at 3,428 cm^-1^ characteristic of hydroxyl group and a weak C-H peak at around 2,915 cm^-1^. The relatively strong absorption peak at around 1,710 cm^-1^ indicated the carbonyl group. 

The absorbance of polysaccharides in the range 1,000-1,200 cm^-1^ was the C-O-C and C-O-H link band positions. The backbone of sugar residues chain in LBP contained 1→6 indican bonds according to periodate oxidation. The results of β-elimination reaction indicated that the chain of polysaccharides and protein was connected by O-linked chemical bond.

*Effects of LBP on locomotor activity in depressive mice*

**Figure 1:**
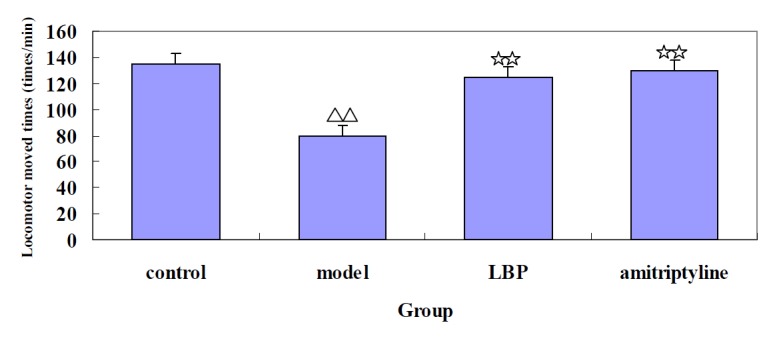
The effect of LBP on locomotor activity in mice. Mice were administered either vehicle, LBP, or amitriptyline before testing. The locomotor moved times were recorded for 4 min. Values are showed as the mean ± SD. With 8-10 mice in each group. Compared with control,^△△^*P *< 0.01; Compared with model,^☆☆^*P *< 0.01

To verify the antidepressant effect of LBP, the locomotor activity was explored using mice in the OFT. We tested the locomotor activity after the administration with LBP. As showed in Figure 1, the results indicated that compared with control, the locomotor activity was significantly decreased in model mice induced by reserpine. After the administration with LBP and amitriptyline, the locomotor activity was significantly increased compared with the model mice.

*Effects of LBP on FST and TST in depressive mice*

**Figure 2:**
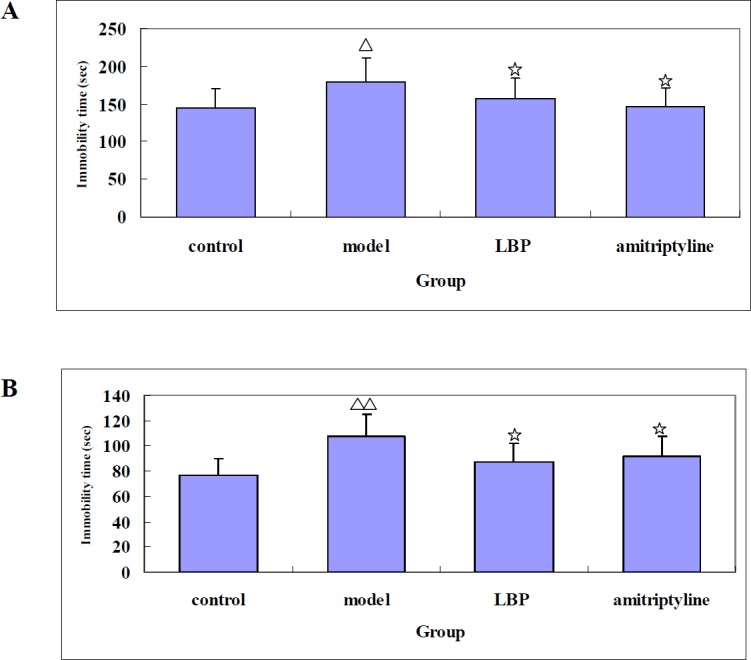
The effect of LBP on the immobility time in mice. (A) the time of forced swimming test (B) the time of tail suspension test. Mice were administered either vehicle, LBP, or amitriptyline before testing. The immobility times of mice in the forced swimming test and tail suspension test were recorded. Values are showed as the mean ±SD. With 8-10 mice in each group. Compared with control, Δ*P*<0.05, ΔΔ*P*<0.01; Compared with model,☆*P *< 0.05

The FST and TST are widely used for the screening of antidepressant drugs. The immobile state of mice present in the FST and TST reflects the despair behavior of the animals. The effects of LBP on the immobility times of the mice in the FST and TST were examined. The FST results showed that compared with the model, LBP could significantly shorten the immobility time of the mice (Figure 2.A). Similar results were observed in the TST, in which LBP significantly reduced the immobility times of the mice. (Figure 2.B).

*Antagonism of hypothermia and ptosis behavior in depressive mice induced by reserpine*

To evaluate the possible involvement of LBP on reserpine-induced hypothermia and ptosis behavior, the effects of LBP on reserpine induced hypothermia and ptosis were determined in the mice. As showed in Table 1, the mice administered with reserpine displayed marked alterations of hypothermia and ptosis. However, administration of LBP for 4 weeks significantly antagonized these symptoms induced by reserpine. At the same time, amitriptyline also reversed all these effects.

*Antioxidant effects of LBP on striatum in depressive mice*

The effects of LBP on the lipid peroxide production in the mice striatum were showed in Table 2 Compared with the control, reserpine induced a significant increase in the TBARS level of the striatum in the model mice (*P *< 0.01). The increased TBARS level induced by reserpine in striatum was inhibited by LBP treatment (*P *< 0.01), whereas, there was no significant difference between the amitriptyline treatment and the depressive mice (model). To explore the ability of LBP on regulating antioxidants in the depressive mice, we investigated the potential effects of LBP against oxidative damage induced on striatum of the mice. As showed in Table 2, compared with the control group, the activities of SOD, GSH-Px, and CAT in the striatum were significantly decreased in model group (*P *< 0.01). LBP treatment for 4 w, compared with the model group, the activities of SOD, GSH-Px, and CAT in striatum were increased significantly. Furthermore, there was no significant difference between the amitriptyline treatment and model.

LBP:*Lycium barbarum.* Polysaccharide.

LBP:*Lycium barbarum. *polysaccharide.

**Figure 3:**
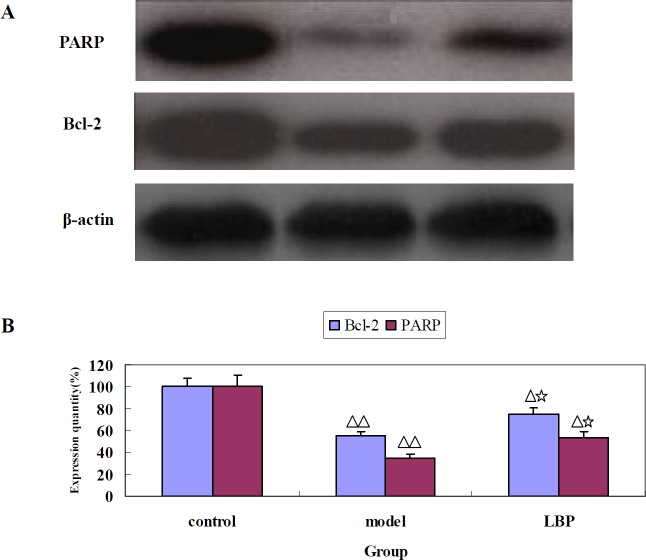
The effect of LBP on the expression of Bcl-2 and PARP in mice striatum. (A) Representative images of Bcl-2 and PARP protein expression detected by western blot. β-actin was used as a control. (B) Quantification of the Bcl-2 and PARP expression. Data are expressed as mean ± SD. from 3 independent experiments. Compared with control, ^Δ^*P *< 0.05, ^ΔΔ^*P *< 0.01; Compared with model

☆*P *< 0.05.

LBP:*Lycium barbarum. *Polysaccharide; PARP:poly (ADP ribose) polymerase.

*Effects of LBP on Bcl-2 and PARP in striatum of depressive mice*

To further investigate the mechanism of LBP on depressive mice, we evaluated the effects of LBP on apoptosis in striatum of depressive mice using western blotting. Bcl-2 and poly (ADP ribose) polymerase (PARP) are major types of regulatory proteins associated with apoptosis. As showed in Figure 3 A, B, Bcl-2 and PARP, two apoptotic suppressors, were markedly decreased after treatment with reserpine, whereas, the protein levels of Bcl-2 and PARP were increased in LBP group (*P *< 0.01), compared with the model group.

## Discussion

Depressive disorder is a severe psychotic disorder that includes symptoms of depressed mood, sleep disturbance, and psychomotor and body weight abnormalities. Its pathogenesis is not yet fully understood. Recently, the increasing interest has been attracted for development and utilization of antidepressant plants because they have fewer side effects and are more economic, especially in developing countries (22). Plant-derived polysaccharides for the management of depression have been reports. For example, fuzi polysaccharide-1 from Aconitum carmichaeli could affect brain-derived neurotrophic factor signaling pathways (23). *Lycium barbarum* has been used as a traditional herbal medicine for thousands of years in China. Its therapeutic activities have been well established in the treatment of such conditions as consumptive disease accompanied by thirst, dizziness, blurred vision, diminished visual acuity, and chronic cough (24, 25). *Lycium barbarum* polysaccharide (LBP), extracted from the traditional Chinese herb *Lycium barbarum*, is found to have bioactivities such as antioxidant (26) and hypoglycemic activities (27). However, very little is known about the effect of *Lycium barbarum* on depressive disorder. In this study, we assessed the antidepressant effects of LBP and its possible mechanisms. Before investigating the effect of LBP on the depressive disorder, the mice were induced by reserpine administration for 15 days. Psychological evaluations cannot be performed directly in mice, and the models are evaluated using only behavioral observations. For example, the most common behavioral outcome measurements are OFT, FST, and TST (28, 29). Locomotor activity is an index of alertness of mental activity as most of the drugs acting on central nervous system (CNS) influence locomotor activity. The OFT was used to assess the effect of the locomotor function of LBP. The results of the study showed that LBP had a very good type of CNS excited activity, as indicated by the increase in the locomotor activity in the mice. An increase in the locomotor function is considered an index of alertness, and the converse is an indication of sedative activity. The FST and TST are classic antidepressant models, which reflect the despair behavior of animals, and can simulate the depressive state. The FST and TST-induced state of immobility in animals is similar to human depression and is amenable to reversal by antidepressant drugs. The present results demonstrated that LBP induces significant antidepressant effects in these models. 

Reserpine is an antihypertensive drug that depletes neuronal storage granules of biogenic amines in the brains of rodents and produces a clinically significant depression-like state (30). Mice become hypothermic, akinetic and ptosis, in response to reserpine. In the reserpine-induced hypothermia antagonism test and ptosis behavior, LBP could effectively inhibit hypothermia and ptosis in the mice induced by reserpine, which were consistent with its clinical effectiveness in the treatment of depression. These results implied that the catecholamine and/or serotonin systems might be involved in the antidepressant-like effect of LBP. The exact mechanism remained to be further discussed.

Recent studies have showed ROS also play a role in the pathogenesis of depressive disorder (12). Furthermore, preclinical studies have demonstrated that the inhibition of oxidative stress may contribute to the therapeutic effects of some antidepressant drugs (20, 31). However, the action mechanism for the antidepressant effect of LBP is still not fully elucidated. Amitriptyline is the most common clinical tricyclic antidepressants, and its pharmacological effects are blocking reuptake of norepinephrine and serotonin in nerve endings. To further investigate the antidepression mechanisms of LBP, the effects of LBP on the oxidative stress of striatum in reserpine-treated mice were studied. Our results showed that the increased TBARS level induced by reserpine in mice striatum was inhibited by LBP treatment (*P *< 0.01). Whereas, there was no significant difference between the amitriptyline treatment and model. This suggested that the antidepressant mechanism of LBP was different from amitriptyline. There is an intrinsic antioxidant defense system in cells for scavenging ROS to prevent cellular damage. Cellular antioxidant defenses usually maintain the ROS levels at concentrations that prevent excess oxidation of cellular molecules (32). Cellular antioxidant defenses are endogenous and are mediated by SOD, GSH-Px, CAT and so on (33). SOD is one of the most important enzymes in the antioxidant defense system. It quenches the superoxide radical by converting it into O_2_ and H_2_O_2_. H_2_O_2_ can be reduced to H_2_O in the presence of GSH-Px or CAT. GSH-Px is an important biomolecule involving in the antioxidant defense system against toxicants. *Lycium barbarum. *constituents including polysaccharides found in the plant extracts are effective as radical scavengers and inhibitors of lipid peroxidation (34-36). In addition, the level of endogenous antioxidant enzymes (SOD, GSH-Px, and CAT) was significantly decreased in the depressive mice induced by reserpine and was also markedly restored by LBP. The results are in line with a previous report showing that LBP could enhance the antioxidant capacities against oxidative stress (37). These results indicate that LBP could modulate the level of ROS and antioxidant enzymes to antagonize depression-induced oxidative stress in mice striatum .

Free radicals are known to induce DNA damage resulting in the activation of poly (ADP ribose) polymerase (PARP) (38), a multifaceted enzyme involved in various cytotoxic mechanisms. Apoptosis is known to involve cleavage of chromosomal DNA into nucleosomal units by caspases, and activation of Bcl-2 family of proteins in response to apoptotic signals such as cell stress, free radical damage etc. It is known that Bcl-2 family proteins play a prominent antiapoptotic role by acting upstream of caspase activation and PARP has a modulatory effect on Bcl-2 expression (39). According to the identified apoptosis of cell signaling, PARP is a nuclear enzyme activated by strand breaks in DNA and implicated in DNA repair, apoptosis, organ dysfunction, or necrosis (40, 41). In addition, it is known that the members of the Bcl-2 protein family are known to be major regulators of cytochrome C release and downstream caspases activation. Thus, Bcl-2 plays a vital role in regulating neurocyte apoptosis (42). In the present study, we found that LBP treatment enhanced anti-apoptotic protein (Bcl-2 and PARP) expression in striatum. This is the first report showing the mechanism of LBP against depressive disorder resulting from apoptosis of striatal neurons mediated by oxidative stress in mice. Importantly, our results demonstrated that anti-oxidative properties of LBP are crucial to the striatum neuroprotection against depressive disorder.

## Conclusion

In conclusion, LBP had antidepressant activity on reserpine-induced mice, and the mechanism was closely correlated with increasing antioxidative properties and decreasing the apoptosis of striatum neuron. These observations could be the background for the further development of LBP as a potential dietary therapeutic agent against depression disorder. 
